# Ethical, Legal, and Social Implications of Newborn Screening in Africa: A Scoping Review

**DOI:** 10.3390/ijns12030046

**Published:** 2026-06-25

**Authors:** Victory Oghenetega Samuel, Abdullahi Adeyinka Adejare, Ushotanefe Useh

**Affiliations:** 1 Lifestyle Diseases Research Entity, Faculty of Health Sciences, North-West University, Mafikeng 2745, South Africa; 53719999@mynwu.ac.za (A.A.A.); ushotanefe.useh@nwu.ac.za (U.U.); 2 Department of Physiology, Faculty of Basic Medical Sciences, College of Medicine, University of Lagos, Lagos 100254, Nigeria

**Keywords:** newborn screening, ethical, legal, and social implications, Africa

## Abstract

Newborn screening initiatives have the potential to mitigate childhood morbidity in Africa, but they also have special ethical, legal, and social implications (ELSI) that are influenced by issues with the health system, cultural diversity, and limited resources. This scoping review explores the ELSI of newborn screening across Africa to identify key challenges, gaps, and future research needs. A systematic search identified 27 peer-reviewed studies published between 2008 and 2025, covering 12 African countries. Data were extracted on study characteristics, disease types, and ELSI dimensions from African Journals Online (AJOL), Scopus, PubMed, Web of Science, and BMJ Journals. Thematic analysis mapped recurring ethical, legal, and social concerns. Most studies examined ethical and social dimensions, while legal frameworks were rarely addressed. South Africa, Tanzania, and Ghana contributed the largest number of publications. Sickle cell disease (52%) and hearing screening (30%) were the dominant foci. Common ethical issues included informed consent, privacy, and justice; legal gaps centered on the absence of data protection and frameworks; and social concerns involved stigma, awareness, and cultural perceptions of hereditary disease. Ethical and social issues dominate NBS discourse in Africa, whereas legal oversight remains limited. To guarantee fair, reliable, and long-lasting newborn screening programs, national policy guidelines, community involvement, and context-specific ethical frameworks must be strengthened.

## 1. Introduction

Newborn screening (NBS), which uses systematic diagnostic procedures to evaluate newborns soon after birth, is a fundamental component of contemporary public health practice. The two main types of newborn screening used worldwide are newborn hearing screening (NHS) and newborn bloodspot screening [1,2]. NBS’s main objective is to make it possible to identify severe but treatable genetic, metabolic, endocrine, and hematological problems long in advance of the emergence of clinical symptoms [3]. Pre-symptomatic detection of major but treatable metabolic, endocrine, hematological, and genetic illnesses is the main objective of newborn screening in order to avert morbidity, death, and developmental dysfunction by prompt intervention [3].

Despite being a routine practice in many affluent nations, the implementation of newborn screening across the African continent remains inconsistent, often limited to just a few trial programs or research endeavors [4]. For instance, Sub-Saharan Africa is home to over 75% of global sickle cell disease (SCD) births, yet newborn bloodspot screening is not widely implemented across the region. Currently, the implementation of NHS across Africa remains limited and largely pilot-based, with routine screening available primarily in urban tertiary hospitals and private healthcare settings in countries such as South Africa, Nigeria, Egypt, and Kenya [5,6]. There are significant differences in access to early hearing screening and intervention because most countries lack national programs, policy frameworks, or adequate audiological infrastructure.

In recent years, newborn screening’s ethical, legal, and social implications (ELSI) have drawn more attention globally [7,8]. Recent advances in genomic technology, the growth of biobanking, and the incorporation of molecular diagnostics into public health initiatives are the reasons for this growing interest. Obtaining legitimate consent, safeguarding individual autonomy, and making sure parents or guardians are fully aware of the implications of test results and any follow-up activities are the main ethical considerations [9]. Legally speaking, discussions usually center on the presence and application of national laws, data governance guidelines, patient privacy, and the extent of regulatory control over biological samples that are stored [10]. Socially, conversations focus on questions of fairness and equity, the possibility of discrimination or stigma, and the significance of maintaining public confidence in screening programs [10].

The ELSI of newborn screening are exceptionally intricate and amplified in African contexts, creating significant implementation challenges. Social stigmas that sustain cultural opposition to screening and legislative loopholes in data protection regimes interact with ethical issues, especially obtaining meaningful informed consent in low-literacy communities. Sickle cell disease (SCD) screening preliminary pilots in Ghana, Nigeria, and South Africa show promise, with initial participation rates ranging from 79 to 92% [11]. However, significant loss-to-follow-up (30–50%) and uneven funding cause prolonged implementation to flounder, exposing systemic infrastructure deficiencies. Furthermore, the deployment of health screening initiatives within under-resourced environments, particularly those lacking comprehensive healthcare provision or resilient infrastructure, frequently presents significant ethical dilemmas concerning justice and equity [12].

The ELSI of newborn screening in Africa have not been well reviewed. Ethically sound, culturally relevant NBS frameworks are hampered by fragmented research in genetics, public health, and bioethics that lack cohesive analysis [13,14]. In order to identify recurring barriers, methodological and geographic gaps, and policy prospects, this scoping study maps ELSI findings across African contexts. In order to give policymakers and practitioners a solid body of evidence, it attempts to consolidate the body of research on consent difficulties in low-literacy contexts, the absence of legal mandates, cultural stigma obstacles, and infrastructure ethics. In the end, this review seeks to steer the creation of context-specific NBS methods that strike a compromise between resource constraints and ethical requirements, facilitating fair, long-term scale-up across various African health systems. A scoping review was preferred in this study as it maps diverse evidence, identifying gaps and themes across complex issues, as we did not intend to answer a specific, narrow question. It was used to identify the extent, range, and nature of available evidence, clarify key concepts, and uncover knowledge gaps without formally evaluating the quality of the included evidence. Unlike systematic reviews, it accommodates varied study designs, capturing broad implications and contextual factors, informing policy and practice in this evolving African context.

## 2. Methods

### 2.1. Eligibility Criteria

This scoping review includes studies focusing on the ethical, legal, and social implications of newborn screening in Africa. Specifically, studies that met the following criteria were included: (1) Studies involving newborns, parents, families, healthcare workers, policymakers, or communities related to newborn screening in African countries; (2) Research focusing on ethical, legal, or social implications of newborn screening including studies addressing issues like consent, equity, privacy, data governance, stigma, access, or cultural beliefs; (3) Studies conducted in one or more African countries, or explicitly focusing on African populations even if conducted elsewhere; (4) Empirical studies (qualitative, quantitative, or mixed methods), policy analyses, or ethical analyses published in peer-reviewed English journals and grey literature such as government or NGO reports if they address ELSI aspects; (5) Studies not earlier than 2008. Studies were excluded if they are (1) conducted entirely outside Africa without any African data, discussion, or population relevance; (2) not involving newborns or not referring to newborn screening (e.g., adult genetic testing, prenatal screening); (3) purely biomedical or technical evaluations of screening accuracy, sensitivity/specificity, or lab techniques without ELSI focus; (4) edi, torials, review articles, commentaries, conference abstracts (unless containing original data), book reviews, or opinion pieces without substantive data or analysis; (5) not published in English language unless they are translatable.

### 2.2. Information Sources

**Figure 1:**
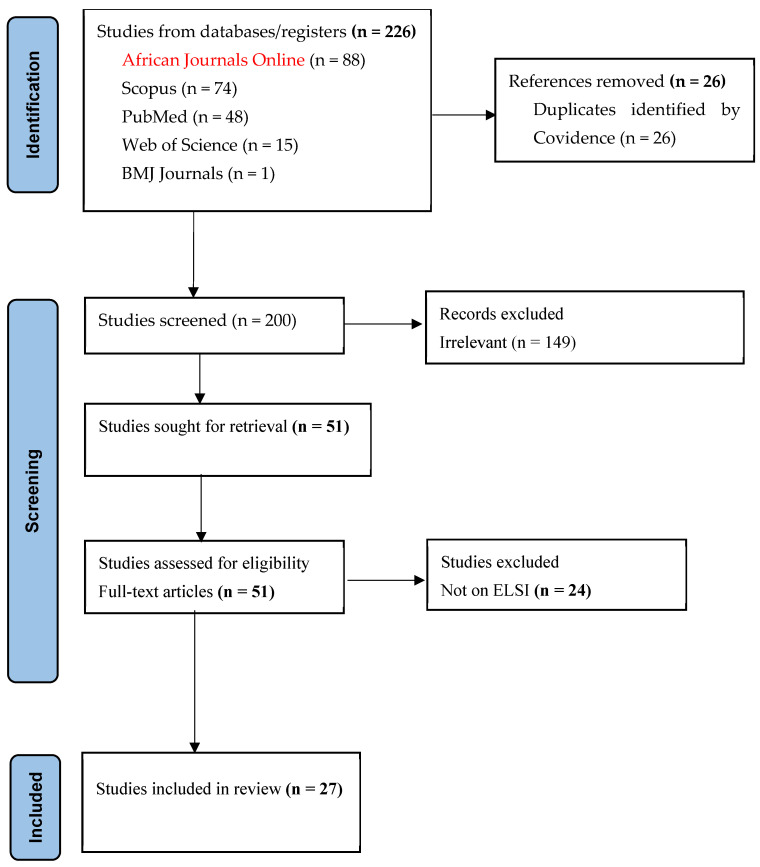
PRISMA-ScR flow diagram of study selection process.

Two independent reviewers, V.O.S. and A.A.A., conducted a systematic search of African Journals Online (AJOL), Scopus, PubMed, Web of Science, and BMJ Journals for studies published between 1 January 2008 and 31 December 2025. They used a combination of Medical Subject Headings (MeSH) and free text terms to identify relevant studies, and also searched the databases using a tailored set of terms. The search was limited to peer-reviewed, English-language human studies. To supplement the database search, the reviewers manually screened references and other sources. The full search strategies are detailed in Figure 1.

### 2.3. Search Strategy

(TITLE-ABS-KEY (“newborn screening” OR “neonatal screening”)) AND (TITLE-ABS-KEY (ethics OR ethical OR legal OR social OR “social implications”)) AND (TITLE-ABS-KEY (Africa OR African)). This was edited to fit into the syntax required for each of the databases included in this study.

### 2.4. Selection Process

The selection of studies for this scoping review was structured according to the PCC framework (Population, Concept, Context) recommended by the Joanna Briggs Institute (JBI). The review followed the PRISMA extension for Scoping Reviews (PRISMA-ScR) guidelines. Screening of studies was carried out by V.O.S. and A.A.A. using the Covidence online software (Veritas Health Innovation Ltd., Melbourne, Australia; https://app.covidence.org/reviews/684524 (accessed on 18 November 2025)). The two researchers initially screened the identified articles based on titles and abstracts, applying pre-defined eligibility criteria. They then reviewed full-text copies of the remaining articles, excluding irrelevant ones. A third researcher (U.U.) mediated any disagreements on inclusion or exclusion. The researchers also examined the references of included articles to identify additional relevant studies. The detailed search process is outlined in Figure 1.

### 2.5. Data Collection Process and Items

Extraction of data was carried out by V.O.S. and A.A.A. independently. The data collection process involved extracting data on the study characteristics (authors, year, country, region), disease studied, key findings, ethical, legal and social concerns or issues addressed. Standardized, independently pretested forms were designed specifically for this purpose. Disagreements on the extracted data were resolved by a third author, U.U.

### 2.6. Reporting Guidelines

The review was reported according to PRISMA extension for Scoping Reviews (PRISMA-ScR) guidelines and registered with the Open Science Framework (OSF): https://osf.io/p5gfc/ (accessed on 18 May 2026). Being a scoping review, a formal quality appraisal of the included studies was not conducted.

### 2.7. Synthesis Methods

We used narrative synthesis in this scoping review to provide a comprehensive overview of the existing literature, highlighting key findings, themes, gaps, and methodological limitations. In the context of newborn screening in Africa, our categorization of the issues was guided by the fact that ethical issues relate to moral principles guiding decision-making, such as autonomy, beneficence, and justice, while social issues focus on the practical, societal impacts, like stigma, access, and cultural influences. For instance, informed consent is an ethical concern, while unequal access to screening services is a social issue reflecting broader healthcare disparities. The method also allows for interpretation and synthesis of complex data, identifying patterns and trends, and informing future research, policy, or practice decisions, particularly when diverse study types are involved. The key findings from each study, including the ethical, legal, and social concerns addressed, were presented in tables. The diseases screened and the countries where the studies were carried out were presented as bar charts. Lastly, the ethical, legal, and social implications themes were presented as a pie chart.

## 3. Results

### 3.1. Study Selection

The database search yielded 226 articles from various sources: African Journals Online (88), Scopus (74), PubMed (48), Web of Science (15), and BMJ Journals (1). After removing 26 duplicates, 200 articles remained. Screening excluded 149 articles that didn’t meet the criteria, such as non-African studies or non-newborn populations. This left 51 articles, and 24 more were excluded as they were not focused on ELSI, leaving 27 articles for inclusion. The process is as illustrated in Figure 1.

### 3.2. Study Characteristics, Key Findings and the ELSI Addressed

A total of 27 studies published between 2008 and 2025 were included, covering 12 African countries. South Africa, Tanzania, and Ghana were the most represented. This is illustrated in Table 1. Ethical and social implications were the most explored dimensions, followed by legal considerations. Findings highlight high acceptability of screening but persistent barriers, including limited resources, workforce shortages, cultural beliefs, and funding gaps. Ethical, legal, and social implications are widely discussed, particularly stigma, consent, and inequities in access. While some regions demonstrate successful pilot programs and positive health outcomes, many studies emphasize the need for sustainable policies, improved infrastructure, public education, and stronger government support to enhance screening implementation and long-term care. The key findings and characteristics of the studies included in this review are summarized in Table 1 below.

### 3.3. Conditions Screened

**Figure 2:**
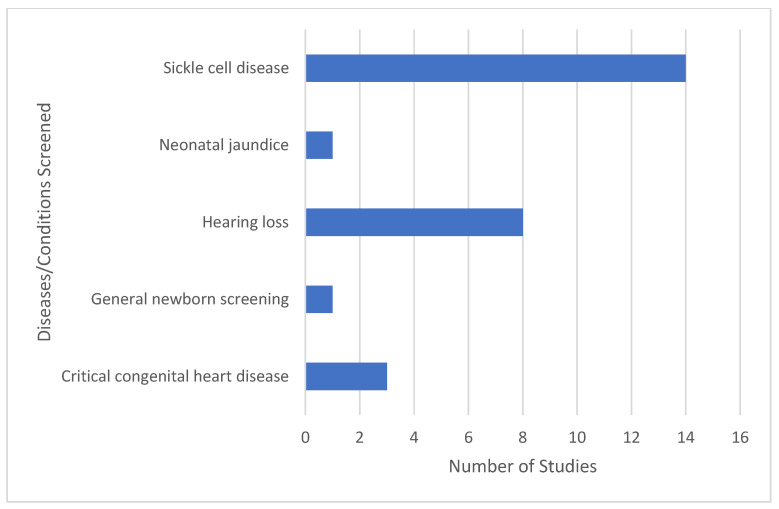
A bar chart illustrating the number of diseases/conditions screened in the review.

Based on the studies included in this review, the studies primarily focused on sickle cell disease (52%), hearing screening (30%), and other congenital or metabolic conditions (18%). The reason for this is not far-fetched; Sub-Saharan Africa alone is home to over 75% of global sickle cell disease (SCD) births. Critical congenital heart disease was reported by three studies, while there was 1 study each that reported neonatal jaundice and general newborn screening. It should be noted that there are other diseases that are screened at neonatal levels that are not included in this review; this review only covers those studies that include or reflect ELSI themes. This is illustrated in Figure 2.

### 3.4. Countries/Regions Included in the Review

**Figure 3:**
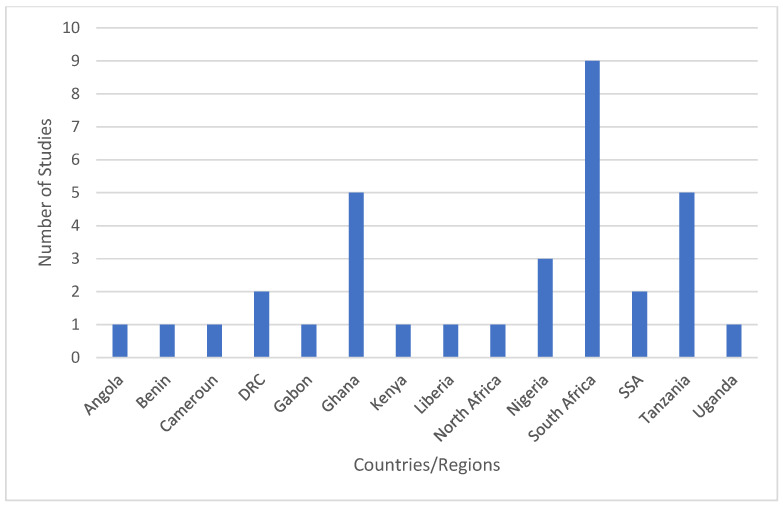
A column chart illustrating the countries and regions included in the review. Key: Democratic Republic of Congo (DRC); Sub-Saharan Africa (SSA).

From the results, South Africa is the leading country with the highest number of studies, followed by Ghana and Tanzania. Other countries, including Angola, Benin, Cameroon, Kenya, and Uganda, show relatively few studies, while broader regions such as Sub-Saharan Africa and North Africa are also included. This is illustrated in Figure 3.

### 3.5. ELSI Themes Addressed

**Figure 4:**
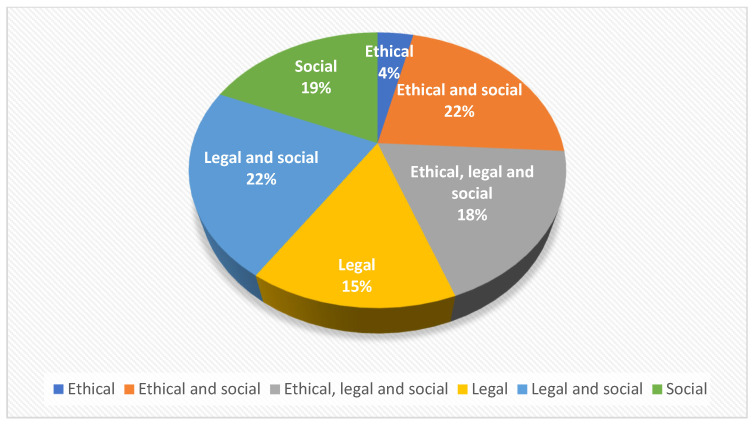
A pie chart illustrating the ELSI themes addressed in this study.

The pie chart shows the distribution of ELSI in newborn screening studies. “Ethical and social” and “Legal and social” issues are the most common, each at 22%, followed by “Social only” (19%), “Ethical, legal and social” (18%), and “Legal” (15%). Purely “ethical” concerns are the least reported (4%), indicating a stronger emphasis on combined implications. This is illustrated in Figure 4.

## 4. Discussion

This scoping review sheds light on the ELSI of newborn screening in Africa. Our review makes it clear that ethical, legal, and social issues are not just side concerns but are at the heart of newborn screening (NBS) in Africa. Even so, these issues are still not fully built into national health policies across many countries. Looking at the 27 studies included in the review, most of them focused heavily on ethical and social questions, showing how deeply community beliefs, levels of health understanding, and unequal access to care shape how screening programs work in real life. Legal and policy matters, however, received less attention, even though they are just as important for making sure these programs are fair, effective, and sustainable over time. Without clear laws and systems of accountability, newborn screening efforts risk being inconsistent and uneven, especially across regions with different resources. The review suggests that while there is growing awareness of the importance of screening, there is still a gap between research findings and actual policy implementation. This gap leaves many countries without a clear direction, making it harder to scale up screening programs in a way that is both ethical and practical. Overall, the findings point to the need for a more balanced approach that gives equal attention to ethical, legal, and social factors when designing and expanding newborn screening systems.

### 4.1. Ethical Dimensions

Ethical concerns in the African context often center on informed consent, autonomy and justice. In many communities, getting truly informed consent is not straightforward. Low literacy levels and limited understanding of genetic conditions make it difficult for parents to fully grasp what screening involves. In addition, decisions about a child’s health are often made within families or communities rather than by individuals alone, which differs from the more individual-focused approach seen in Western healthcare systems [9,11]. Moreover, parents may consent to screening without fully understanding the implications of a positive result or the requirements for lifelong management of certain genetic disorders [40]. The issue of equity and justice also features prominently. Many African countries lack universal access to newborn screening and follow-up care, meaning that even when disorders are detected early, appropriate treatment may not be available or affordable [3,12]. This creates ethical concerns about distributive justice, whether it is fair to screen for conditions without guaranteeing the necessary medical support. Additionally, questions of beneficence and non-maleficence arise when screening expands faster than the healthcare system’s ability to manage positive cases [41]. Another emerging ethical concern is data stewardship. As some NBS programs begin to store residual bloodspots for potential future research, parents often remain unaware of how these samples are used [8]. The absence of standardized policies on biobanking and secondary data use risks eroding public trust and violating ethical principles of transparency and respect for persons.

A key debate in newborn screening is whether it should be based on parental consent or made mandatory. Consent-based approaches respect the rights of parents to make informed decisions about their child’s care, but they may lead to some babies missing out on early diagnosis and treatment. On the other hand, mandatory screening aims to protect public health by ensuring that all newborns are tested, but it can limit parental choice and raise ethical concerns. In African settings, this debate is even more complex due to limited healthcare resources and lower levels of awareness about genetic conditions. In some cases, consent processes may not work well because parents do not fully understand the information they are given. At the same time, making screening compulsory could worsen existing inequalities if follow-up care is not available to everyone. The review suggests that a balanced approach is needed, one that takes local realities into account and involves communities in decision-making. Lessons from countries like the United States and Canada show that even in well-resourced settings, newborn screening systems continue to evolve, with ongoing efforts to improve coordination and reduce differences between regions. These examples highlight the importance of building systems that are both flexible and fair, while also learning from global experiences to improve local practices.

### 4.2. Legal and Policy Considerations

Legal frameworks for newborn screening in Africa are still in their infancy. Few countries have enacted comprehensive legislation regulating genetic testing, data storage, or biobanking [10]. This regulatory vacuum exposes patients to potential misuse of genetic information and limits accountability among implementing institutions. For example, while South Africa and Ghana have initiated steps toward establishing ethical oversight committees, many nations still operate NBS programs without clearly defined policies on consent documentation, result disclosure, or long-term data retention [42]. In addition, data protection and privacy laws remain fragmented or poorly enforced across the continent. Only a handful of countries, such as Kenya and Nigeria, have enacted digital data protection acts that could theoretically extend to genetic data, but these laws rarely address biological specimens explicitly [43]. This creates a gray area regarding the ownership and control of genetic information, raising serious concerns about confidentiality and cross-border data sharing.

Weak legal frameworks also impede regional harmonization. Given that many African populations are genetically diverse and mobile, cross-border collaboration is crucial for building large, representative databases that can enhance diagnostic accuracy. However, without aligned policies on genetic data governance, such collaborations face significant ethical and legal barriers [7]. In Africa, mandatory newborn screening raises ethical concerns about autonomy and informed consent, particularly in resource-constrained settings. Legal frameworks are often lacking or inadequate, risking rights violations. Politically, governments must balance public health benefits with individual freedoms, prioritizing vulnerable populations. Cultural sensitivities and community engagement are crucial to ensure equitable, respectful implementation that aligns with local values and promotes health justice. The study by [36] recommended integrating newborn screening services into national health care systems to improve coverage, accessibility, and affordability. This, according to the authors, would help sustain program implementation and provide comprehensive care services. In the African context, unfortunately, making newborn screening mandatory by integrating it into national health care systems raises ethical concerns about autonomy and informed consent. Legally, there are no frameworks for such arrangements, and politically, governments of most African countries are not ready to provide the necessary funding support to ensure actualization and sustainability.

### 4.3. Social and Cultural Dimensions

Social implications of NBS in Africa are deeply intertwined with cultural beliefs, stigma, and trust in medical systems. Hereditary conditions such as sickle cell disease (SCD) are often misunderstood and stigmatized, leading to social exclusion and marital discrimination [11,15]. In some settings, parents may conceal positive results to avoid community judgment, thereby undermining early intervention efforts. These patterns highlight the need for culturally sensitive counseling and public education that contextualizes genetic conditions within local belief systems. The role of community engagement is therefore vital. Studies from Ghana, Nigeria, and Tanzania reveal that community-driven awareness campaigns significantly improve participation and retention rates in NBS programs [31,37,38]. Engaging traditional leaders and community health workers can help dispel myths and foster trust in screening interventions. Moreover, persistent socioeconomic inequities compound these social challenges. Screening initiatives are typically concentrated in urban tertiary hospitals, excluding large rural populations who often experience the highest disease burden [12,43]. This geographic disparity not only limits coverage but also reinforces existing inequities in access to diagnostic and treatment services.

### 4.4. Integration and Future Directions

The collective findings from this review reveal that while the feasibility and acceptability of newborn screening (NBS) have been demonstrated in several African countries, the integration of ethical, legal, and social considerations into policy and practice remains fragmented. The studies analyzed consistently underscored that ethical reflections such as informed consent and equity are often discussed in isolation from the legal and social mechanisms that enable their implementation. This separation has contributed to inconsistencies in how NBS is governed, communicated, and sustained across national contexts. Future studies should move beyond feasibility assessments to explore how NBS programs can be institutionalized within broader health system reforms—supported by clear legislation, interdisciplinary collaboration, and community-driven approaches. Only through such integrative efforts can NBS achieve its full potential as an equitable and ethically sound public health intervention across Africa.

In conclusion, newborn screening in Africa sits at the intersection of ethics, law, and society, and progress in this area depends on addressing all three together. The review shows that while there is strong interest in expanding screening programs, major gaps remain in policy, legal protection, and social support systems. Ethical concerns about consent, fairness, and data use must be addressed in ways that respect local cultures and realities. Legal systems need to be strengthened to protect individuals and support cooperation between countries. At the same time, social barriers such as stigma, prejudices, lack of awareness, and unequal access to care must be actively tackled through education and community engagement. A prominent theme is the limited healthcare infrastructure and workforce, with many countries struggling with inadequate staffing, equipment shortages, and budget constraints, hindering effective implementation of screening programs. The overall message is that newborn screening cannot succeed as a purely medical intervention. It requires a broader, more integrated approach that considers people’s lived experiences, protects their rights, and ensures that benefits are shared fairly. By learning from both local experiences and global practices, African countries can develop screening systems that are not only effective but also ethical, inclusive, and sustainable over the long term. Integration into national healthcare systems is seen as key to improving coverage, accessibility, and affordability, with local efforts to sustain program implementation offering encouraging models for other low-resource settings.

### 4.5. Limitations

This scoping review has several limitations: it covers only 12 of 54 African countries, limiting geographical representation; the 17-year review period may not capture long-term trends; and exclusion of non-English articles may omit relevant data, potentially biasing findings and reducing generalizability across the continent’s diverse contexts. Expanding the scope of future reviews to include more African countries, extending the review period, and incorporating non-English articles to enhance geographical representation, capture long-term trends, and reduce bias would increase the generalizability of findings across Africa’s diverse contexts.

## 5. Conclusions

This scoping review reveals ELSI as a fundamental barrier to NBS implementation in Africa. Ethical consent challenges, legal policy gaps, and social stigma demand context-specific solutions. Targeted research in underrepresented regions, coupled with legislated mandates and community engagement, can enable equitable NBS scale-up, substantially reducing childhood morbidity from SCD and other screened conditions continent-wide.

## Figures and Tables

**Table 1:** Characteristics and key findings from the studies included in this review.

S/N	Author(s)	Country/ Region	Disease Studied	Key Findings	ELSI Addressed
1.	Ebomoyi [15]	Sub-Saharan Africa (SSA)	Sickle cell disease	(1) Ethical issues: Low priority for SCD mass screening in SSA communities; traditional healers provide ineffective management. (2) Bone Marrow Transplantation (BMT) and gene therapy practiced in developed nations; Sub-Saharan African patients airlifted for critical care. Legal ramifications of BMT complications require prudent practice but limited physician liability. (3) Social stigma of SCD newborns: spirit children ostracized. Workforce/infrastructure shortages worsen psychosocial effects. (4) Inadequate health funding drives high SCD prevalence in SSA.	Ethical, legal and social
2.	Van Niekerk, Cullis [16]	South Africa (Western Cape)	Critical congenital heart disease (CCHD)	Pulse oximetry screening was highly acceptable to staff/parents. Without staff shortages, routine pre-discharge implementation is feasible, yielding low false positives, minimal errors, and no cardiology burden.	Ethical and social
3.	Swanepoel and Almec [17]	South Africa (Gauteng)	Hearing loss	99% of mothers desired postnatal hearing screening, and there was a high acceptance of hearing aids.	Ethical and social
4.	Rahimy, Gangbo [18]	Benin	Sickle cell disease	Among the consecutive pregnant women studied (about 3000), 79.5% at-risk pregnant women requested SCD testing; 85.2% positive newborns were enrolled; more than 80% were still retained after 5 years.	Ethical and social
5.	Phanguphangu, Kgare [19]	South Africa (Eastern Cape)	Hearing loss	Limited, uneven Early Hearing Detection and Intervention (EHDI) resource distribution negatively impacts service provision.	Legal
6.	Munung, Kamga [20]	Cameroon, Ghana, and Tanzania.	Sickle cell disease	The results show a general preference for newborn screening for SCD over prenatal and premarital/preconception testing due to simpler decision-making and lower stigmatization risk.	Ethical, legal and social
7.	Mombo, Makosso [21]	Gabon (Koula-Moutou)	Sickle cell disease	The acceptance of SCD screening for newborns is unrelated to disease knowledge or maternal hemoglobin status. Barriers stem from education and culture, not awareness.	Ethical and social
8.	Meyer and Swanepoel de [22]	South Africa	Hearing	Nationally, 53% of private sector obstetric units offered some newborn hearing screening, with 14% providing universal screening. The most common approaches were screening on select days of the week (18%) or on request (18%). The primary barrier to implementation was the omission of newborn hearing screening from maternity birthing packages.	Legal
9.	Khoza-Shangase, Kanji [23]	South Africa (Gauteng and North-West)	Hearing	No formal, standardised EHDI implementation exists across primary, secondary, and tertiary care due to insufficient knowledge, equipment shortages, budget constraints, and human resource limitations. Regardless of care level or resource allocation, the Health Professions Council of South Africa (HPCSA) 2007 EHDI recommendations remain unfeasible without addressing barriers and mandating NHS.	Ethical, legal and social
10.	Khoza-Shangase, Kanji [24]	South Africa	Hearing	EHDI implementation gaps persist in South Africa, with 83.7% of participants conducting newborn hearing screening but over half using targeted rather than universal approaches. Capacity-demand challenges hinder success, as 60% of audiologists maintain NHS should be audiologist-only with minimal task-shifting. No standardized protocols exist alongside persistent budget allocation issues.	Legal and social
11.	Katamea, Mukuku [25]	Congo	Sickle cell disease	77.7% of participants demonstrated good knowledge of SCD as a hereditary blood disorder, while NBS acceptability stood at 84.5%. Age (*p*-value = 0.002), sex (*p*-value = 0.025), and religion (*p*-value < 0.001) showed significant associations with NBS acceptability.	Legal and social
12.	Harbinson and Khoza-Shangase [26]	South Africa	Hearing	Efficient and comprehensive newborn screening proves unsuccessful within hours of birth under current staffing profiles and practices but succeeds more at the Midwife Obstetric Unit (MOU) three-day assessment clinic.	Ethical and social
13.	Govender, Ghuman [27]	South Africa (KZN)	Critical congenital heart disease (CCHD)	The main barriers were inadequate staffing and infrastructure.	Legal and social
14.	Bukini, Mbekenga [28]	Tanzania	Sickle cell disease	Analysis of families’ and nurses’ experiences reveals the gendered relations that undergird childcare and how those relations influence the quality of care the child may potentially receive. These findings highlight the need to study SCD’s social implications in Africa to improve regional patient care. Gendered dynamics in childcare directly influence healthcare delivery and family support systems	Social
15.	Bakari, Wolski [29]	Ghana (Kumasi)	Neonatal Jaundice	The Bili-Ruler demonstrated high acceptability and feasibility among mothers and families for newborn jaundice monitoring. In total, 98% of mothers reported using it, with 90.8% applying it three or more days during the first week postpartum and 89.8% using it more than twice daily. These usage patterns indicate strong potential for integration into routine postnatal care in resource-limited settings.	Social
16.	Archer, Inusa [30]	Angola, Democratic Republic of Congo, Ghana, Liberia, Nigeria and Tanzania	Sickle cell disease	Analysis of implementation challenges revealed four primary themes: governance (such as deploying overcommitted clinical staff for NBS), technical aspects (including operational process design), cultural factors (like varying knowledge among community-based staff), and financial issues (where external funding may limit government involvement). Key learnings highlighted factors contributing to long-term NBS program sustainability. While establishing enduring NBS programs improves health outcomes for populations with SCD, initial implementation in Africa does not guarantee sustainability.	Ethical, legal and social
17.	Anie, Treadwell [31]	Ghana (Kumasi)	Sickle cell disease	Workshop findings emphasized enhanced SCD knowledge among the public most especially the youths as crucial, alongside the vital role of elders, religious, and traditional leaders as stakeholders, as well as the government’s goals to reduce SCD births.	Ethical and social
18.	Kanji, Naude [5]	South Africa	Hearing loss	While agreement existed on optimal timing and format for information delivery, consent processes varied between private and public sectors, with inconsistent information provision. Focus groups underscored the nuances of obtaining true informed consent, distinct from implied consent or choice, highlighting its clinical complexity. Findings emphasized accessible, culturally sensitive information as an essential for parental autonomy and informed decision-making.	Ethical
19.	Krotoski, Namaste [32]	Middle East and North Africa	General	For countries without national NBS programs, the steering committee prioritized congenital hypothyroidism (CH) due to its high prevalence, available screening methods, and cost-effective intervention, forming a training working group for educational materials. Common barriers include shortages of trained professionals, financial/political support, geographic isolation affecting access and reagent transport, lack of mandatory policies, and inadequate databases for linking screened children to long-term care. To date, there are no concrete measures to control the burden of genetic disorders in the region, such as systematic neonatal screening, which are made in NA, and initiatives have been practically abandoned.	Legal and social
20.	Makani, Soka [33]	Tanzania	Sickle cell disease	A pilot programme for the implementation of NBS for SCD is planned to start in 2015, with the aim of integrating the NBS policy into the reproductive and child health (RCH) programme.	Legal
21.	Nnodu, Adegoke [34]	Nigeria	Sickle cell disease	Respondents demonstrated good knowledge of SCD as an inherited blood disorder, with 86% (*n* = 1119) supporting NBS. Statistically significant relationships existed between NBS support and age (*p*-value = 0.03), educational status (*p*-value = 0.00), and religion (*p*-value = 0.00).	Social
22.	Nnodu, Okeke [11]	Sub-Sahara Africa	Sickle cell disease	(1) Barriers to NBS in SSA are lack of government support/political will, low public awareness/maternal education, poor access to screening facilities, diagnostic lab supply shortages/stock-outs (2) Facilitators to NBS in SSA are government ownership (policy/funding/import waivers), stakeholder involvement in planning/workflows, NBS acceptability, integration into existing services (3) The Consortium on Newborn Screening in Africa (CONSA) Initiative was launched in 2017 by the American Society of Hematology for SCD NBS feasibility in Nigeria, Tanzania, Uganda, Liberia, Ghana, Kenya, Zambia; first networks 2020 targeting 10,000–16,000 newborns/country.	Ethical, legal, and social
23	Orimbo, Awandu [35]	Kenya	Sickle cell disease	Only maternal age and occupation were significantly associated with the acceptability of newborn screening for sickle cell disease. Mothers aged 25−34 years were 3 times less likely to accept newborn screening for sickle cell disease than younger mothers under 25 years (OR = 0.33; 95%CI = 0.13–0.86; *p* = 0.024).	Social
24.	Bukini, Nkya [36]	Tanzania	Sickle cell disease	A key area for strengthening involves fully integrating newborn screening services into national health care systems to improve coverage, accessibility, and affordability. Although sickle cell disease screening coverage remains low, local efforts to sustain program implementation and comprehensive care services offer encouraging models for other low-resource settings.	Legal and social
25.	Inusa, Anie [37]	Nigeria (Kaduna state)	Sickle cell disease	Using the 10 Getting to Outcomes (GTO) accountability questions provided a structured framework to identify implementation strengths and weaknesses. For example, a major communication gap emerged between policymakers and user groups.	Legal and social
26.	Green, Mathur [38]	Uganda	Sickle cell disease	Healthcare providers recognized the need to expand SCD care through regional hub clinics supported by local pediatric facilities. Parents showed reluctance for text-based newborn health communication due to unfamiliarity and misinterpretation risks. Advocacy for public/private resources addressing medical, counseling, and social needs remains essential, with follow-up for screen-positive cases best delivered via home-based telephone, volunteer, or village health worker visits. Newborn screening programs must extend beyond labs to overcome implementation barriers and improve child health outcomes.	Social
27.	Adadey, Quaye [39]	Ghana	Hearing loss	Early testing for the GJB2-R143W variant enables timely detection of hearing impairment and facilitates medical and social services to enhance affected individuals’ quality of life. Adoption of this test as part of Ghana’s universal newborn hearing screening (UNHS) program is recommended.	Legal

## Data Availability

The original contributions presented in this study are included in the article. Further inquiries can be directed to the corresponding author.

## References

[1] Therrell B.L., Padilla C.D., Borrajo G.J.C., Khneisser I., Schielen P.C.J.I., Knight-Madden J., Malherbe H.L., Kase M. (2024). Current Status of Newborn Bloodspot Screening Worldwide 2024: A Comprehensive Review of Recent Activities (2020–2023). Int. J. Neonatal Screen..

[2] Wen C., Zhao X., Li Y., Yu Y., Cheng X., Li X., Deng K., Yuan X., Huang L. (2022). A systematic review of newborn and childhood hearing screening around the world: Comparison and quality assessment of guidelines. BMC Pediatr..

[3] Therrell B.L., Padilla C.D., Loeber J.G., Kneisser I., Saadallah A., Borrajo G.J., Adams J. (2015). Current status of newborn screening worldwide: 2015. Semin. Perinatol..

[4] Wesonga R.M., Awe O.I. (2022). An Assessment of Traditional and Genomic Screening in Newborns and their Applicability for Africa. Inform. Med. Unlocked.

[5] Kanji A., Naudé A., Moore J. (2025). Balancing Sound Decisions: Exploring Informed Consent Practices and Perspectives in Newborn Hearing Screening Programs. Am. J. Audiol..

[6] Louw P.H., Odendaal T., Ramma L. (2024). Mapping neonatal hearing screening services in Cape Town metro: A situational analysis. Afr. J. Prim. Health Care Fam. Med..

[7] Cabello J.F., Novoa F., Huff H.V., Colombo M. (2021). Expanded Newborn Screening and Genomic Sequencing in Latin America and the Resulting Social Justice and Ethical Considerations. Int. J. Neonatal Screen..

[8] Sénécal K., Unim B., Knoppers B.M. (2018). Newborn screening programs: Next generation ethical and social issues. OBM Genet..

[9] Ulph F., Bennett R. (2022). Psychological and Ethical Challenges of Introducing Whole Genome Sequencing into Routine Newborn Screening: Lessons Learned from Existing Newborn Screening. New Bioeth..

[10] Anderson R., Rothwell E., Botkin J.R. (2011). Newborn Screening: Ethical, legal, and social implications. Annu. Rev. Nurs. Res..

[11] Nnodu O.E., Okeke C.O., Isa H.A. (2024). Newborn screening initiatives for sickle cell disease in Africa. Hematol. Am. Soc. Hematol. Educ. Program.

[12] Abimbola S., Pai M. (2020). Will global health survive its decolonisation?. Lancet.

[13] Twum S., Fosu K., Felder R.A., Sarpong K.A. (2023). Bridging the gaps in newborn screening programmes: Challenges and opportunities to detect haemoglobinopathies in Africa. Afr. J. Lab. Med..

[14] Satekge T., Okesina A., Anetor J., Erasmus R. (2025). The status of newborn screening in Africa: Situation analysis, future plans and call to action. Afr. J. Lab. Med..

[15] Ebomoyi E.W. (2015). Ethical, legal, social, and financial implications of neonatal screening for sickle cell anaemia in Sub-Sahara Africa in the age of genomic science. Int. J. Med. Eng. Inform..

[16] Van Niekerk A.M., Cullis R.M., Linley L.L., Zühlke L. (2016). Feasibility of Pulse Oximetry Pre-discharge Screening Implementation for detecting Critical Congenital heart Lesions in newborns in a secondary level maternity hospital in the Western Cape, South Africa: The ‘POPSICLe’ study. S. Afr. Med. J..

[17] Swanepoel D., Almec N. (2008). Maternal views on infant hearing loss and early intervention in a South African community. Int. J. Audiol..

[18] Rahimy M.C., Gangbo A., Ahouignan G., Alihonou E. (2009). Newborn screening for sickle cell disease in the Republic of Benin. J. Clin. Pathol..

[19] Phanguphangu M., Kgare K., Flynn A., Kotelana S., Mfeketo S., Njiva S. (2024). Availability of resources for paediatric hearing care in a South African province. Afr. J. Prim. Health Care Fam. Med..

[20] Munung N.S., Kamga K.K., Treadwell M.J., Dennis-Antwi J., Anie K.A., Bukini D., Makani J., Wonkam A. (2024). Perceptions and preferences for genetic testing for sickle cell disease or trait: A qualitative study in Cameroon, Ghana and Tanzania. Eur. J. Hum. Genet..

[21] Mombo L.E., Makosso L.K., Bisseye C., Mbacky K., Setchell J.M., Edou A. (2021). Acceptability of neonatal sickle cell disease screening among parturient women at the Paul Moukambi Regional Hospital in rural Eastern Gabon, Central Africa. Afr. J. Reprod. Health.

[22] Meyer M.E., de Swanepoel W. (2011). Newborn hearing screening in the private health care sector—A national survey. S. Afr. Med. J..

[23] Khoza-Shangase K., Kanji A., Petrocchi-Bartal L., Farr K. (2017). Infant hearing screening in a developing-country context: Status in two South African provinces. S. Afr. J. Child Health.

[24] Khoza-Shangase K., Kanji A., Ismail F. (2021). What are the current practices employed by audiologists in early hearing detection and intervention in the South African healthcare context?. Int. J. Pediatr. Otorhinolaryngol..

[25] Katamea T., Mukuku O., Mpoy C.W., Mutombo A.K., Luboya O.N., Wembonyama S.O. (2022). Factors Associated with Acceptability of Newborn Screening for Sickle Cell Disease in Lubumbashi City, Democratic Republic of the Congo. Glob. J. Med. Pharm. Biomed. Updat..

[26] Khoza-Shangase K., Harbinson S. (2015). Evaluation of universal newborn hearing screening in South African primary care. Afr. J. Prim. Health Care Fam. Med..

[27] Govender S., Ghuman M., Coutsoudis A. (2018). An investigation into the challenges and limitations of implementing universal pulse oximetry screening for critical congenital heart disease in asymptomatic newborns. SA Heart.

[28] Bukini D., Mbekenga C., Nkya S., Malasa L., McCurdy S., Manji K., Makani J., Parker M. (2021). Influence of gender norms in relation to child’s quality of care: Follow-up of families of children with SCD identified through NBS in Tanzania. J. Community Genet..

[29] Bakari A., Wolski A.V., Otoo B., Amoah R., Nakua E.K., Jacovetty J., Kaselitz E., Compton S.D., Moyer C.A. (2025). Using a Hand-Held Icterometer to Screen for Neonatal Jaundice: Validation, Feasibility, and Acceptability of the Bili-RulerTM in Kumasi, Ghana. Int. J. Environ. Res. Public Health.

[30] Archer N.M., Inusa B., Makani J., Nkya S., Tshilolo L., Tubman V.N., McGann P.T., Ambrose E.E., Henrich N., Spector J. (2022). Enablers and barriers to newborn screening for sickle cell disease in Africa: Results from a qualitative study involving programmes in six countries. BMJ Open.

[31] Anie K.A., Treadwell M.J., Grant A.M., Dennis-Antwi J.A., Asafo M.K., Lamptey M.E., Ojodu J., Yusuf C., Otaigbe A., Ohene-Frempong K. (2016). Community engagement to inform the development of a sickle cell counselor training and certification program in Ghana. J. Community Genet..

[32] Krotoski D., Namaste S., Raouf R.K., El Nekhely I., Hindi-Alexander M., Engelson G., Hanson J.W., Howell R.R. (2009). Conference report: Second conference of the Middle East and North Africa newborn screening initiative: Partnerships for sustainable newborn screening infrastructure and research opportunities. Genet. Med..

[33] Makani J., Soka D., Rwezaula S., Krag M., Mghamba J., Ramaiya K., Cox S.E., Grosse S.D. (2015). Health policy for sickle cell disease in Africa: Experience from Tanzania on interventions to reduce under-five mortality. Trop. Med. Int. Health.

[34] Nnodu O.E., Adegoke S.A., Ezenwosu O.U., Emodi I.I., Ugwu N.I., Ohiaeri C.N., Brown B.J., Olaniyi J.A., Isa H., Okeke C.C. (2018). A Multi-centre Survey of Acceptability of Newborn Screening for Sickle Cell Disease in Nigeria. Cureus.

[35] Orimbo J., Awandu S.S., Muhonja F., Owili P., Omondi D. (2025). High acceptability of newborn screening for sickle cell disease among post-natal mothers in Western Kenya. PLoS ONE.

[36] Bukini D., Nkya S., McCurdy S., Mbekenga C., Manji K., Parker M., Makani J. (2021). Perspectives on Building Sustainable Newborn Screening Programs for Sickle Cell Disease: Experience from Tanzania. Int. J. Neonatal Screen..

[37] Inusa B.P., Anie K.A., Lamont A., Dogara L.G., Ojo B., Ijei I., Atoyebi W., Gwani L., Gani E., Hsu L. (2018). Utilising the ‘Getting to Outcomes®’ Framework in Community Engagement for Development and Implementation of Sickle Cell Disease Newborn Screening in Kaduna State, Nigeria. Int. J. Neonatal Screen..

[38] Green N.S., Mathur S., Kiguli S., Makani J., Fashakin V., LaRussa P., Lyimo M., Abrams E.J., Mulumba L., Mupere E. (2016). Family, Community, and Health System Considerations for Reducing the Burden of Pediatric Sickle Cell Disease in Uganda Through Newborn Screening. Glob. Pediatr. Health.

[39] Adadey S.M., Quaye O., Amedofu G.K., Awandare G.A., Wonkam A. (2021). Screening for GJB2-R143W-Associated Hearing Impairment: Implications for Health Policy and Practice in Ghana. Public Health Genom..

[40] Botkin J.R., Belmont J.W., Berg J.S., Berkman B.E., Bombard Y., Holm I.A., Levy H.P., Ormond K.E., Saal H.M., Spinner N.B. (2015). Points to Consider: Ethical, Legal, and Psychosocial Implications of Genetic Testing in Children and Adolescents. Am. J. Hum. Genet..

[41] Yong S.E.F., Wong M.L., Voo T.C. (2022). Screening is not always healthy: An ethical analysis of health screening packages in Singapore. BMC Med. Ethics.

[42] Hitzeroth H.W., Niehaus C.E., Brill D.C. (1995). Phenylketonuria in South-Africa—A Report on the Status-Quo. S. Afr. Med. J..

[43] Juma I., Faturoti B. (2025). Enforcing data privacy in Kenya and Nigeria: Towards an African approach to regulatory practice. Int. Rev. Law Comput. Technol..

